# Novel Identified Circular Transcript of RCAN2, circ-RCAN2, Shows Deviated Expression Pattern in Pig Reperfused Infarcted Myocardium and Hypoxic Porcine Cardiac Progenitor Cells In Vitro

**DOI:** 10.3390/ijms22031390

**Published:** 2021-01-30

**Authors:** Julia Mester-Tonczar, Patrick Einzinger, Johannes Winkler, Nina Kastner, Andreas Spannbauer, Katrin Zlabinger, Denise Traxler, Dominika Lukovic, Ena Hasimbegovic, Georg Goliasch, Noemi Pavo, Mariann Gyöngyösi

**Affiliations:** 1 Department of Internal Medicine II, Division of Cardiology, Medical University of Vienna, 1090 Vienna, Austria; julia.mester-tonczar@meduniwien.ac.at (J.M.-T.); johannes.winkler@univie.ac.at (J.W.); nina.kastner@meduniwien.ac.at (N.K.); andreas.spannbauer@meduniwien.ac.at (A.S.); katrin.zlabinger@meduniwien.ac.at (K.Z.); denise.traxler-weidenauer@meduniwien.ac.at (D.T.); dominika.lukovic@meduniwien.ac.at (D.L.); n1542442@students.meduniwien.ac.at (E.H.); georg.goliasch@meduniwien.ac.at (G.G.); noemi.pavo@meduniwien.ac.at (N.P.); 2 Institute of Information Systems Engineering, Research Unit of Information and Software Engineering, Vienna University of Technology, 1040 Vienna, Austria; patrick.einzinger@tuwien.ac.at

**Keywords:** circRNA, circ-RCAN2, AMI, porcine model of myocardial infarction, pCPCs

## Abstract

Circular RNAs (circRNAs) are crucial in gene regulatory networks and disease development, yet circRNA expression in myocardial infarction (MI) is poorly understood. Here, we harvested myocardium samples from domestic pigs 3 days after closed-chest reperfused MI or sham surgery. Cardiac circRNAs were identified by RNA-sequencing of rRNA-depleted RNA from infarcted and healthy myocardium tissue samples. Bioinformatics analysis was performed using the CIRIfull and KNIFE algorithms, and circRNAs identified with both algorithms were subjected to differential expression (DE) analysis and validation by qPCR. Circ-RCAN2 and circ-C12orf29 expressions were significantly downregulated in infarcted tissue compared to healthy pig heart. Sanger sequencing was performed to identify the backsplice junctions of circular transcripts. Finally, we compared the expressions of circ-C12orf29 and circ-RCAN2 between porcine cardiac progenitor cells (pCPCs) that were incubated in a hypoxia chamber for different time periods versus normoxic pCPCs. Circ-C12orf29 did not show significant DE in vitro, whereas circ-RCAN2 exhibited significant ischemia-time-dependent upregulation in hypoxic pCPCs. Overall, our results revealed novel cardiac circRNAs with DE patterns in pCPCs, and in infarcted and healthy myocardium. Circ-RCAN2 exhibited differential regulation by myocardial infarction in vivo and by hypoxia in vitro. These results will improve our understanding of circRNA regulation during acute MI.

## 1. Introduction

Circular RNAs (circRNAs) are long non-coding RNAs (lncRNAs) characterized by a closed-loop structure that lacks 3′ poly(A) tails and 5′ cap structures [1]. Since their initial discovery in 1976 in viroids (i.e., circular single-stranded RNA pathogens of higher plants) [2], circRNAs have long been thought to be splicing by-products or generated through splicing errors [3,4]. The development of high-throughput sequencing has enabled increasing circRNA research. Over the past decade, next-generation sequencing (NGS) has led to the identification of thousands of circRNAs in humans, nematodes, mice, and pigs [5,6,7,8], and their functions and roles in diseases have been investigated in neurologic disorders, cancer, and cardiovascular diseases (CVD) [9,10,11]. CircRNAs reportedly act as microRNA (miRNA) sponges by inhibiting miRNA-mRNA binding [12,13], and can also interact with RNA-binding proteins [14,15]. Despite their classification as non-coding molecules, some circRNAs can be translated in a cap-independent manner [16,17]. However, the exact mechanism of circRNA translation is not yet fully understood. Furthermore, circRNAs show tissue- and development-specific expression patterns [18,19]. Investigating circRNA expression patterns is essential for improving our understanding of their functions in diseases. Recent studies indicate imperative roles of circRNAs in cellular processes and disease development [20,21]. A 2019 study demonstrated circFOXP1 involvement in the transcriptional preservation of stem cell identity, and showed that silencing circFOXP1 reduces human mesenchymal stem cell (huMSC) growth and proliferation [22].

In CVD, circFndc3b modulates cardiac function after myocardial infarction (MI) via FUS/VEGF signaling [15]. Additionally, the circRNAs ZFAS1 and CDR1as may act as novel whole blood biomarkers that can distinguish between acute MI (AMI) and control patients [23]. Moreover, a 2019 study identified circNfix as a super-enhancer-regulated circRNA involved in cardiac regeneration after MI [8]. However, this area has not yet been sufficiently studied and there remains a need to identify altered circRNA expression patterns in AMI and to elaborate their molecular functions, which may lead to the development of new diagnostic and therapeutic treatment methods. In the present study, we performed RNA sequencing analysis (RNA-seq) and used two state-of-the-art algorithms to identify circRNA expressions in infarcted and control pig hearts. We then performed qPCR with divergent primers to validate our RNA-seq data in tissue samples. Finally, we further analyzed the expression patterns of the two most promising circRNAs, circ-RCAN2 and circ-C12orf29, in porcine cardiac progenitor cells (pCPCs) under hypoxic and normoxic conditions. Our findings will advance our knowledge of circRNAs in relation to AMI, and improve our understanding of their roles in CVD.

## 2. Results

### 2.1. Bioinformatics Analysis of Cardiac-Related circRNAs in Healthy and Infarcted Pig Hearts

**Figure 1:**
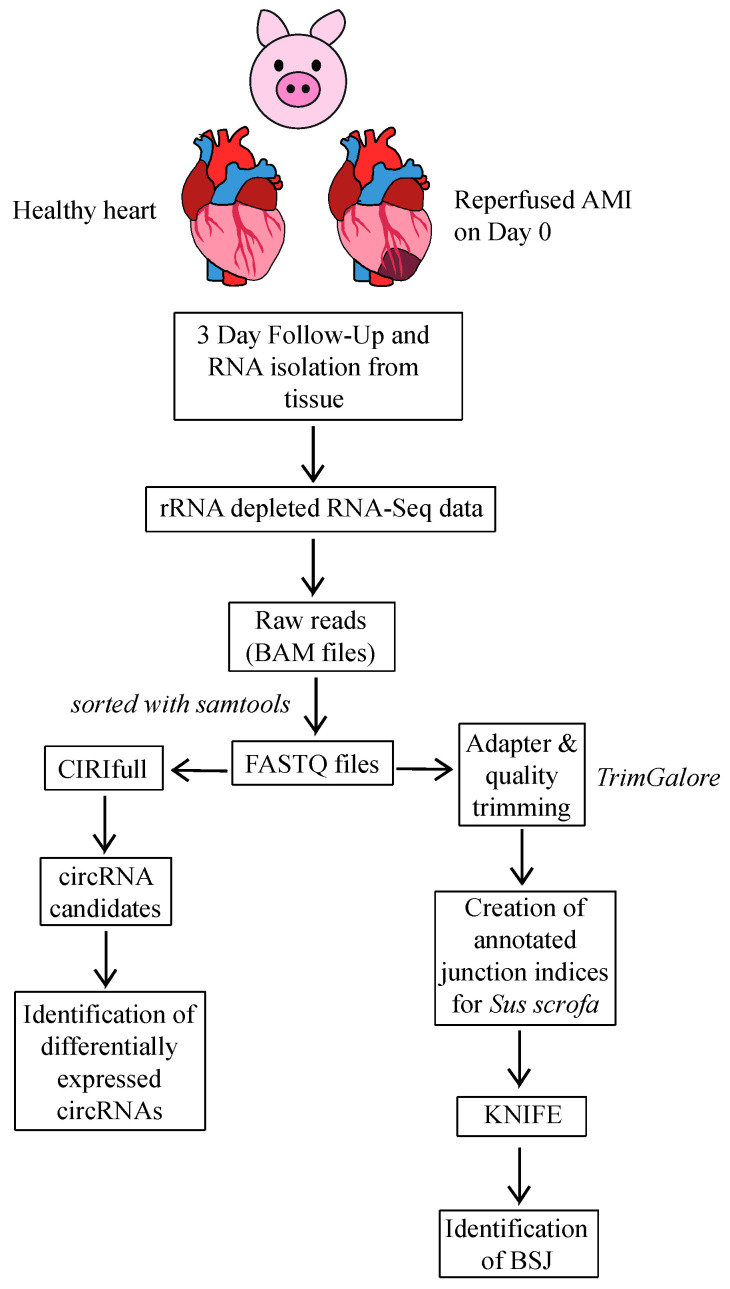
Identification of circRNAs in pig hearts. Study design and bioinformatics pipeline for the identification of circRNAs using reads of rRNA-depleted RNA from pig hearts at three days after reperfused acute myocardial infarction (AMI) (*n* = 3) or sham operation (healthy pig hearts) (*n* = 3).

We analyzed the expression of cardiac circRNAs in infarcted and healthy myocardium tissue of domestic pigs, three days after closed-chest reperfused MI (or sham operation). To identify cardiac circRNAs in rRNA-depleted RNA of infarcted and healthy porcine myocardium, we used the CIRIfull [24] and KNIFE [25] algorithms. KNIFE is pre-packaged with annotated junction indices for human, mouse, rat, and drosophila, but not for *Sus scrofa*. Therefore, we created a new junction index using the Ensemble reference genome *Sus scrofa* 11.1 and annotations (downloaded from iGenome, https://support.illumina.com/sequencing/sequencing_software/igenome.html). Transcriptomic sequences were extracted with gffread, and ribosomal sequences were acquired from NCBI. From these input data, we prepared Bowtie indices, as well as linear and scrambled junction indices, following the instructions at https://github.com/lindaszabo/KNIFE/tree/master/createJunctionIndex. CIRIfull and KNIFE were used independently from each other to ensure correct backsplice junction (BSJ) identification in circular transcripts and to eliminate false positives [26]. Figure 1 shows the study design and bioinformatics pipeline.

CIRIfull identified 5487 fully reconstructed circRNAs, while KNIFE identified the BSJs of 8668 circRNAs. We additionally analyzed the types of circRNAs expressed in porcine hearts, and found that most of the candidates were exonic.

**Figure 2:**
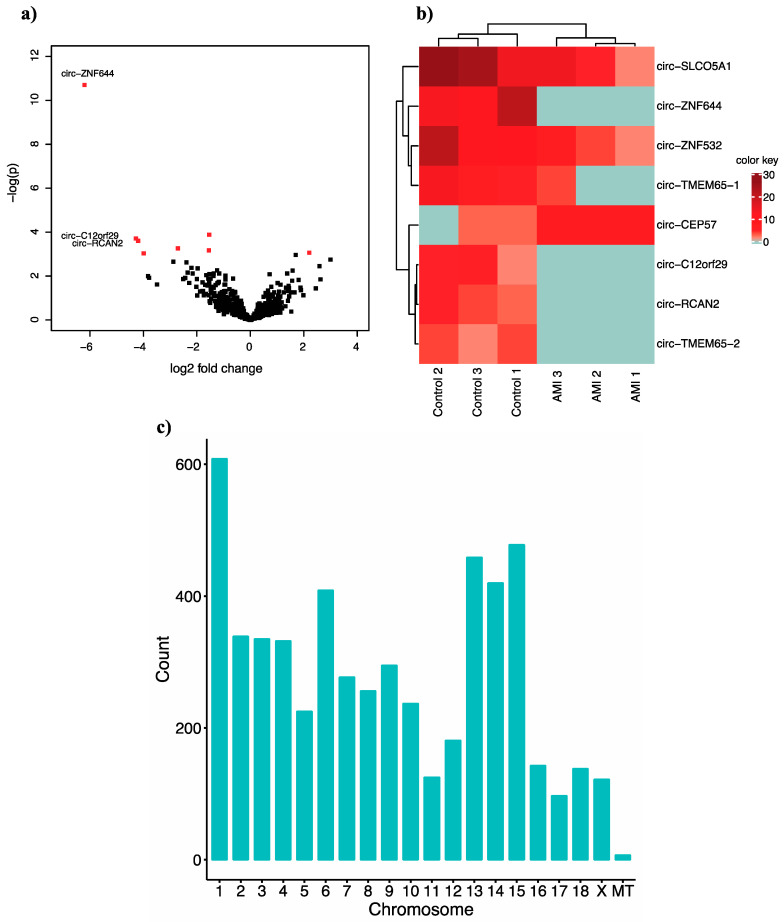
Regulation of circRNAs in infarcted (AMI) and healthy (control) pig hearts. (**a**) Volcano plot shows identified circRNAs. Red dots indicate significantly dysregulated circRNAs. (**b**) Heat-map shows the eight circRNAs with deviating expression patterns between AMI and control tissue. (**c**) Distribution of circRNAs among chromosomes.

Using the fully reconstructed circRNAs detected by CIRIfull, we performed differential expression (DE) analysis with DESeq2. This revealed significant DE of eight circRNAs in infarcted pig hearts compared to healthy controls. In Figure 2A, a volcano plot shows the *p* values and log2 fold changes. In Figure 2B, a heat-map displays the read counts of our RNA-seq data. Figure 2C illustrates the circRNA distribution on the chromosomes, indicating that the most circRNAs in pig hearts were located on chromosome 1.

### 2.2. Validation of Our RNA-Seq Data

**Figure 3:**
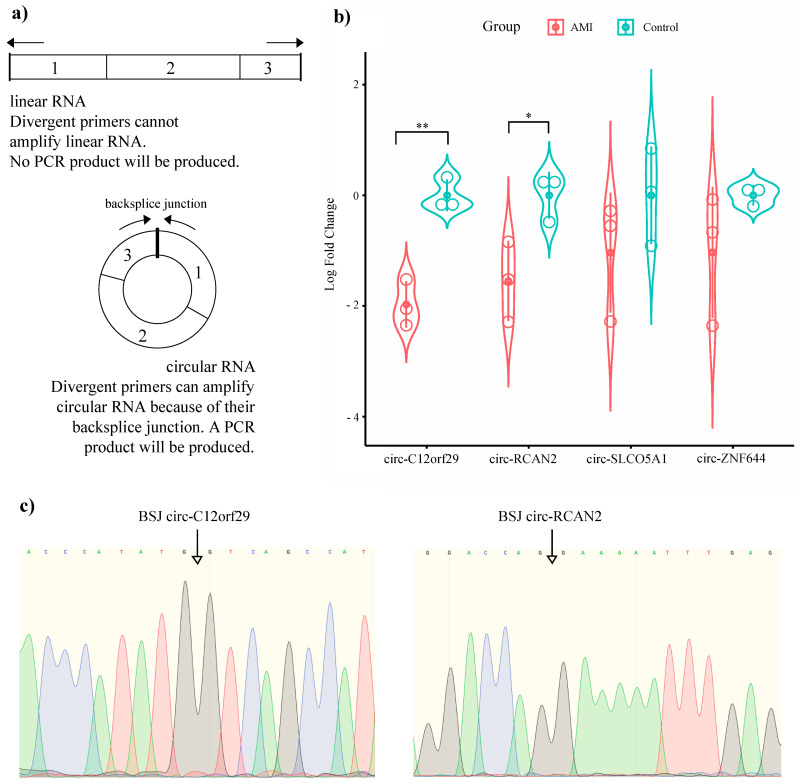
Validation of the RNA-seq data in porcine heart tissue. (**a**) Divergent primers were used to amplify the backsplice junction of the circRNAs. Arrows show the directions of the forward/reverse primers. Numbers inside the boxes indicate the exons. (**b**) Differential expression analysis of our four circRNAs between reperfused AMI and healthy heart using qPCR. * *p* < 0.05, ** *p* < 0.01. Error bars show standard deviation (SD). (**c**) Sanger sequencing chromatogram shows the backsplice junction.

Among the total identified circRNAs, we selected the circRNAs that were significantly dysregulated between the two study groups for validation in porcine heart tissue using quantitative PCR (qPCR). For these eight circRNAs, we designed divergent primers spanning the BSJ (Figure 3A). The qPCR data validated four of the circRNAs that showed significantly deviating expression patterns between AMI and healthy pig myocardium in our RNA-seq data: circ-ZNF644 (*p* = 2.25 × 10^−5^), circ-SLCO5A1 (*p* = 0.021), circ-RCAN2 (*p* = 0.027), and circ-C12orf29 (*p* = 0.024) (Figure 3B). Of these four circRNAs, only two showed significant downregulation in reperfused AMI compared to control tissue based on qPCR: circ-C12orf29 (*p* = 0.004) and circ-RCAN2 (*p* = 0.044) (Figure 3B). After qPCR, Sanger sequencing was performed, confirming the BSJ of the circRNAs (Figure 3C).

### 2.3. CircRNA Expression Pattern in Hypoxic and Normoxic pCPCs In Vitro

Based on our qPCR results, we selected circ-C12orf29 and circ-RCAN2 for further investigation in pCPCs under normoxic and hypoxic conditions in vitro. The genomic locus of circ-C12orf29 in pigs is on chromosome 5. To our knowledge, no further function has been described for circC12orf29. Our bioinformatics analysis revealed that the genomic locus of circ-RCAN2 in pigs lies on chromosome 7 on the regulator of the calcineurin 2 gene (RCAN2), and that the BSJ is located at 7:41003826-41008498.

**Figure 4:**
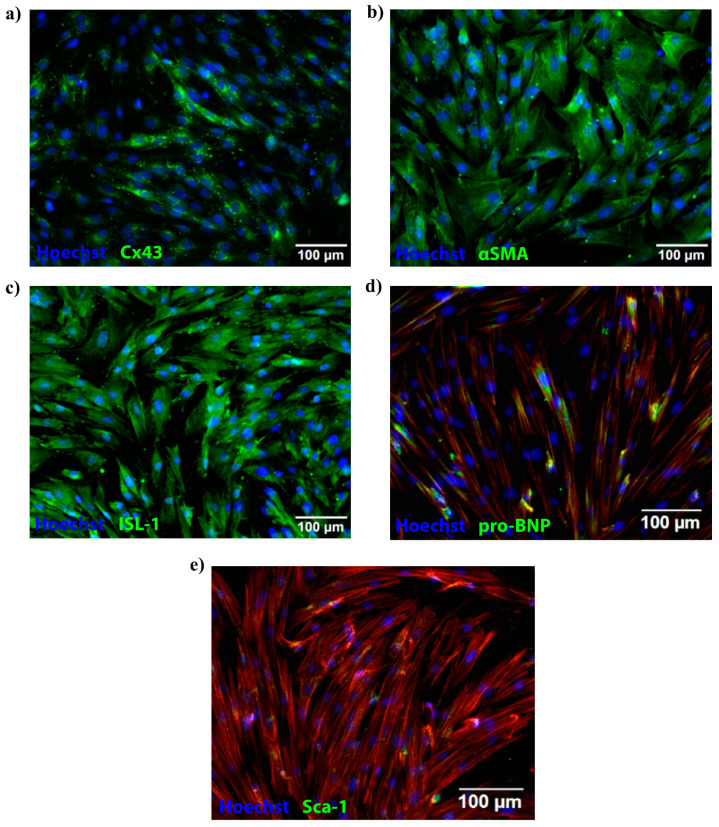
Characterization of pCPCs using immunofluorescence staining. Results showed positive expression of Cx43 (**a**), αSMA (**b**), and pro-BNP (**d**), and of markers associated with cardiac progenitor cells, such as Isl-1 (**c**) and Sca-1 (**e**). In panels (**d**,**e**), the cytoplasm was counterstained with Phalloidin. Nuclei were stained with Hoechst.

We isolated pCPCs as previously described [27], and performed immunofluorescence staining. Our results showed positive expression of connexin-43 (Cx43) (Figure 4a, green), alpha smooth muscle actin (αSMA) (Figure 4b, green), islet-1 (Isl-1) (Figure 4c, green), pro-BNP (Figure 4d, green), and stem cells antigen-1 (Sca-1) (Figure 4e, green), confirming successful isolation of Sca-1^+^, and Isl1^+^ pCPCs.

**Figure 5:**
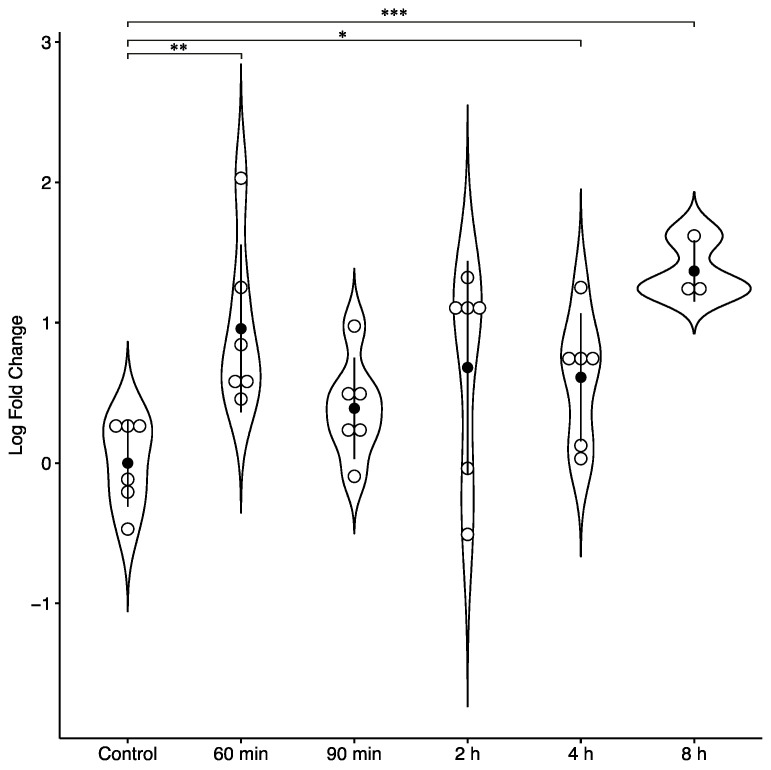
Differential expression analysis of circ-RCAN2 in hypoxic and normoxic pCPCs in vitro. * *p* < 0.05, ** *p* < 0.01, *** *p* < 0.001. Error bars show SD.

In our in vitro experiment, pCPCs were cultured either under normoxic conditions, or in a hypoxic chamber for 60 min, 90 min, 2 h, 4 h, or 8 h without further normoxic culturing of the cells, thus without mimicking reperfusion in vivo. Then, we measured the expressions of circ-C12orf29 and circ-RCAN2. Circ-C12orf29 did not exhibit any significant up- or downregulation in vitro. However, circ-RCAN2 showed a deviated expression pattern in pCPCs, with upregulation in hypoxic pCPCs compared to in normoxic cells (Figure 5). In contrast with the circ-RCAN2 downregulation observed in reperfused AMI tissue at 3 days after infarction, constant hypoxia of pCPCs led to significant circ-RCAN2 upregulation, which was dependent on ischemia time, compared to control cells (Figure 5).

## 3. Discussion

In our present study, we revealed novel cardiac circRNAs in a pig model using deep RNA-sequencing paired with state-of-the-art bioinformatics algorithms. We also created annotated junction indices for *Sus scrofa* for the KNIFE algorithm, as KNIFE only had annotated junction indices for mouse, rat, human, and drosophila. Our expression pattern analysis revealed decreased circ-RCAN2 expression in porcine reperfused ischemic myocardium compared to healthy porcine heart tissue. On the other hand, within a constant hypoxic milieu, pCPCs showed a time-dependent increase in circ-RCAN2 expression. These results suggest that ischemia time and restoration of normoxic conditions play a role in circRNA regulation.

The literature includes only scarce information regarding circRNA expression patters in infarcted myocardium; therefore, here we focused on the detection of novel circRNAs linked to MI in pig hearts. We found that the genomic locus of circ-RCAN2 in pigs lies on chromosome 7 on RCAN2, and the BSJ is located at 7:41003826-41008498. Furthermore, we demonstrated that circ-RCAN2 was significantly downregulated in a porcine model of reperfused MI compared to healthy hearts. To our knowledge, this is the first description of a circular transcript of RCAN2.

The RCAN gene family members RCAN1, RCAN2, and RCAN3—also known as adapt78 [28], DSCR1 [29], and MCIP1 [30]—are inhibitors of the Ca^2+^-activated protein phosphatase calcineurin [29,31,32]. Calcineurin is crucial for cardiac remodeling [33], and is present at notably high levels in excitatory cells, such as cardiomyocytes, that exhibit frequent and rapid changes in cytoplasmic calcium, indicating that such cells require high calcineurin activity [34]. Upregulated expression levels of RCAN homologs have been reported in various diseases, including cardiac hypertrophy and diabetes [35].

One member of the RCAN gene family, RCAN1, has been investigated for its role in ischemia/reperfusion (I/R) injury [36]. In an in vitro study, Parra et al. found that RCAN1-depleted cardiomyocytes were more susceptible to I/R damage [33]. However, our present bioinformatics analysis did not reveal a circular transcript of RCAN1. One possible explanation is that no circular transcript of RCAN1 exists. In this study, we detected circ-RCAN2. Little is presently known regarding the possible function of RCAN2 in the heart and, to our knowledge, no circular transcript of RCAN2 has previously been identified. We found that circ-RCAN2 was significantly downregulated in the reperfused infarcted tissue after 3 days. In contrast, in pCPCs incubated under constant hypoxia (without mimicking reperfusion) for different time periods, we observed time-dependent significant upregulation of circ-RCAN2 compared to in normoxic cells. This may be related to the fact that the pigs underwent 90-min occlusion of the left anterior descending coronary artery (LAD), followed by reperfusion, and tissue was harvested 3 days later. In contrast, in the in vitro experiment, we applied constant hypoxic conditions to our pCPCs for different time periods, without subsequent normoxia. This difference might explain the biphasic expression pattern of circ-RCAN2, and emphasizes the importance of reperfusion and ischemia time in AMI, and the differences between in vivo and in vitro conditions.

Our study has several limitations. One is the low number of animals. However, this was a pilot study, and the results are the first documentation of the existence of circ-RCAN2 and its dysregulation in infarcted cardiac tissue in a translational large animal experiment. Additionally, in our experiments, we harvested the transmural myocardial area for analysis at 3 days post-infarction, since the local inflammation and edema peaks at 3 days post-infarction onset. Despite the well-known heterogeneity of transmural myocardial ischemia, we collected transmural tissue samples because it is not possible to macroscopically separate subendocardial tissue from the mid-cardiac and subepicardial tissues, and a transmural section of the infarcted heart mirrors the complexity of the global pathophysiology processes and similarities to human conditions. In accordance with our previous findings regarding the intrinsic remote conditioning of ischemia-nonaffected regions after ischemia/reperfusion [37], we used non-infarcted animals as controls.

Although thousands of circRNAs have been described in the mammalian heart [7], it remains largely unknown which circRNAs may be linked to AMI or hypoxia. CircRNAs can possess gene regulatory functions, reportedly acting as miRNA sponges [12,13] or interacting with RNA-binding proteins (RBPs) [38,39]. Additionally, they show the potential to be used as diagnostic or therapeutic biomarkers in diseases, such as cancer and CVD [40,41,42]. Here, we investigated circRNA expression in porcine heart tissue to unravel novel circRNAs associated with AMI. To our knowledge, our results constitute the first reported detection of a circular transcript of RCAN2, and we further show that circ-RCAN2 is important in infarcted tissue and hypoxic pCPCs in vitro.

## 4. Materials and Methods

### 4.1. Animal Study

This study included six female domestic pigs (*Sus scrofa domesticus*), which were 6 months old and had weights ranging between 25–30 kg at the time of the experiment. These pigs were randomized to either the AMI (*n* = 3) or sham-operated control (*n* = 3) group. After overnight fasting, the animals were anesthetized via intramuscular injection using 12 mg/kg ketamine hydrochloride, 1 mg/kg xylazine, and 0.04 mg/kg atropine. Then, coronary angiography was performed using a 6F guiding catheter (Terumo Medical Corporation, Somerset, New Jersey, USA). For pigs in the AMI group, a balloon catheter (Maverick XL Monorail Balloon Catheter, Boston Scientific) was inserted into the LAD after the first diagonal branch. Then, reperfused AMI was induced by balloon occlusion of the mid LAD at 5 atm for 90 min, followed by balloon deflation and reperfusion. The occlusion was confirmed by control angiography. Three days after AMI or sham procedure, all animals were euthanized under general anesthesia using 10 mL intravenous saturated KCl (10%).

### 4.2. Tissue Sample Collection

During autopsy, we collected tissue samples (150–250 mg) from the transmural infarcted area of the pig hearts (distal anterior tissue, distal to the LAD occlusion site), and from the same location of the left ventricle from healthy pig hearts. In the early phase of infarction, the infarcted tissue was characterized by edema and inflammation, peaking at day 2–3 post onset. For that reason, we chose day 3 for tissue collection. Tissue samples were stored at −80 °C in RNAlater (Thermo Fisher Scientific, Waltham, MA, USA).

### 4.3. RNA Extraction from Porcine Heart Tissue

Tissue samples were thawed at room temperature, and then 25–30 mg of tissue was cut and placed in Precellys CK28 tubes containing Qiazol Lysis buffer (Qiagen, Hilden, Germany). These tissue samples were disrupted and homogenized at room temperature using the 5000:3×20 - 20 s wait program on the Precellys 24 (Bertin, Rockville, MD, USA). Next, the supernatant was transferred into new 2-mL tubes (Eppendorf, Hamburg, Germany) and 200 µL chloroform was added. This mixture was incubated at room temperature for 2 min, and then the tubes were centrifuged at 11.600× *g* at 4 °C for 15 min. The upper aqueous phase was collected and transferred to 2-mL tubes (Eppendorf), and total RNA was isolated using the miRNeasy Mini kit (Qiagen, Hilden, Germany), including a DNase I digestion, following the manufacturer’s protocol. Total RNA quality and quantity were assessed using the Nanodrop One (Thermo Fisher Scientific).

### 4.4. RNA Sequencing of circRNAs

Isolated total RNA was sequenced using the Illumina NextSeq550 sequencing approach. We performed mRNA enrichment using the NEBNext rRNA depletion kit (New England Biolabs, Ipswich, MA, USA). No poly(A) enrichment was performed, to enable accurate circRNA sequencing. The sequencing samples had a mean depth of 140 million paired-end (150 bp) reads. Library preparation and sequencing were performed at the VBCF NGS Unit (www.viennabiocenter.org/facilities).

### 4.5. Bioinformatics

The raw sequencing reads (BAM files) were sorted using samtools, and then converted to FASTQ files using bedtools. Since we had paired-end data, this resulted in two files per sample (with ending R1 and R2). We analyzed our data using two algorithms for circular RNA identification: CIRI-full and KNIFE. Further analyses were performed using the results of CIRI-full, but we checked whether the circular RNA candidates were also identified using KNIFE. Circular RNA candidates identified by both algorithms are far less likely to be false positives, as demonstrated by Hansen et al. [26]. When using the KNIFE algorithm, we trimmed the reads with Trim Galore (default values). On the other hand, CIRI-full requires reads of equal length, and thus we had to use the untrimmed reads. For both algorithms, quality control was performed using FastQC. As further input for the two algorithms, we used reference genomes and annotation files from iGenomes (Ensembl *Sus Scrofa* 11.1). CIRI-full requires prior alignment using the Burrows-Wheeler Alignment Tool (bwa mem), which we performed with option T = 19 (i.e., alignment with a score < 19 is skipped in the output). For KNIFE, we used the recommended values of 13 for junction overlap (because the reads were paired-end), and 100 for the “ntrim” parameter (because the original read length was 150, and the recommended value is 2/3 of this).

### 4.6. Primers

CircRNA primers were designed in accordance with the results of our bioinformatics analyses. All circRNA primers were designed to amplify the backsplice junction. The primers were purified by HPLC, and were purchased from Microsynth (Balgach, Switzerland). The primer sequences are listed in the Supplementary Material 1, Table S1. We used β-actin and hypoxanthine guanine phosphoribosyltransferase (HPRT) as housekeeping genes in porcine heart tissue, and HPRT as a housekeeping gene in pCPCs.

### 4.7. cDNA Synthesis

We performed cDNA synthesis using the QuantiTect Reverse Transcription kit (Qiagen, Hilden, Germany) following the manufacturer’s protocol.

### 4.8. Real-Time Quantitative PCR Analysis

Experiments were each performed twice. For tissue sample analysis, the circRNA expression levels were normalized to the geometric mean of two housekeeping genes: β-actin and HPRT. DE analysis was performed using the ΔΔCt method. We performed qPCR analyses of pig heart tissue in duplicate with a standard curve. For the pCPC analysis, the circRNA expression levels were normalized to HPRT. We performed qPCR analyses of pCPCs in triplicate, and the primers were diluted with RNase-free H_2_O to a final concentration of 10 µM. No-template controls (run in duplicate) served as control wells. The PCR plates were sealed using transparent PCR foil, and qPCR was run using Quantstudio 5 (Thermo Fisher Scientific, Waltham, MA, USA).

### 4.9. Sanger Sequencing

To confirm our sequencing results and the head-to-tail splicing of the selected circRNAs, Sanger sequencing was performed by Microsynth (Balgach, Switzerland) using our designed circRNA primers.

### 4.10. Porcine Cardiac Progenitor Cells

Porcine cardiac progenitor cells were isolated from pig hearts, and qPCR was performed to assess the relative gene expressions of ISL-1, SCA-1, c-Kit, and BNP, as previously described [27]. To further evaluate the expression of progenitor cell markers, such as islet-1 (Isl-1) and stem cell antigen (Sca-1), we also performed immunofluorescence staining. The cells were cultured in a 1:1 ratio of M199 and Dulbecco’s modified Eagle’s medium (DMEM) (Sigma Aldrich, St. Louis, MO, USA), containing 10% fetal bovine serum (FBS, Biochrom Ltd., Cambridge, UK) and 1% pen-strep. Cells were grown to 90% confluence, and then seeded in 96-well plates containing fresh medium and cultivated overnight in a CO_2_ incubator at 37 °C. The next day, the medium was removed, and the cells were washed with 1× D-PBS (Sigma Aldrich) and then fixed with 4% paraformaldehyde (Carl Roth GmbH, Karlsruhe, Germany) in 1× D-PBS for 15 min. Then, the paraformaldehyde was removed, and 1× PBS (Sigma Aldrich) was added to each well. The cells were incubated at room temperature for 5 min, and then PBS was removed and each well was washed twice with 1× D-PBS. The primary antibodies were diluted following the manufacturer’s protocol, with permeabilization buffer comprising 3% non-fat dry milk and 0.1% Triton X-100 (both from Sigma Aldrich). The primary antibodies were added (50 µL per well), followed by a 2-h incubation at room temperature, after which the primary antibodies were aspirated, and each well was washed with 1× D-PBS. Next, the secondary antibodies were diluted with permeabilization buffer following the manufacturer’s protocol, and added to the wells, followed by a 2-h incubation at room temperature protected from light. Finally, the cells were counterstained using Hoechst (1:5000 in D-PBS, Sigma Aldrich) and Phalloidin (1:40 in PBS, Thermo Fisher Scientific). Immunofluorescence staining was analyzed using an Olympus IX83 Inverted Microscope with the cellSens imaging software (Olympus, Tokyo, Japan). The primary and secondary antibodies, including their dilutions, are listed in the Supplementary materials, Table S2.

### 4.11. Hypoxic and Normoxic Conditions of pCPCs

Next, we investigated circRNA expression in pCPCs under normoxic and hypoxic conditions. We cultured pCPCs in a 1:1 ratio of M199 and DMEM (Sigma Aldrich) with 10% FBS and 1% pen-strep, in 6-well plates, with incubation at 37 °C. The media was changed every 2 days until the cells reached the 90% confluence, at which time the medium was changed to serum-free medium. After an overnight incubation at 37 °C, the cells were placed in a hypoxia incubator chamber (Stemcell Technologies, Vancouver, Canada) for different time periods (60 min, 90 min, 2 h, 4 h, and 8 h). Control cells were not put in the hypoxia incubator chamber (normoxic conditions). To harvest the cells, the medium was removed, the wells were washed with 1× D-PBS, and 350 µL RLT buffer containing β-mercaptoethanol (1:100, Sigma Aldrich) was added to each well. Finally, the cells were transferred into 2-mL tubes (Eppendorf) and stored at −80 °C until further use.

### 4.12. RNA Extraction from pCPCs

Cells in RLT buffer were thawed at room temperature until the solution was completely liquid, and then transferred into QIAshredder tubes (Qiagen). These tubes were then centrifuged at 11.600× *g* for 2 min at 4 °C, and the flow-through was transferred into 2-mL collection tubes (Qiagen). RNA was isolated using the QIAcube and the miRNeasy Mini kit (Qiagen). RNA quantity and quality was assessed using the Nanodrop One (Thermo Fisher Scientific).

### 4.13. Statistics

We performed differential expression analysis of the RNA-seq data (cases vs. controls) using the package DESeq2 [43], which applies a model based on the negative binomial distribution. Prior to the analysis, we filtered transcripts with low expression by requiring counts to be over 3 in more than two of the samples. Results with a *p* value of <0.05 were considered significant. We performed differential expression analyses of the qPCR results for cardiac tissue (cases vs. control) and pCPCs (hypoxia of 60 min, 90 min, 2 h, 4 h, or 8 h vs. normoxia) using the ΔΔCt method, with unequal variance t-tests applied to the log fold changes.

### 4.14. Ethics Approval

Animal handling procedures were conducted in accordance with the “Guide for The Care and Use of Laboratory Animals“ [44], and were approved by the ethical review committee of the University of Kaposvar (SOI/31/26-11/2014).

## 5. Conclusions

In conclusion, here we identified and validated novel circRNAs, and examined their variable expression patterns in infarcted and healthy pig hearts. We confirmed the presence of circ-RCAN2 in pCPCs in vitro, and revealed important differences in deregulation under hypoxic conditions both in vivo and in vitro. Our findings elucidate an interesting new target for MI.

## Supplementary Materials

Supplementary materials can be found at https://www.mdpi.com/1422-0067/22/3/1390/s1.

## Abbreviations

AMI	Acute myocardial infarction
BSJ	Backsplice junction
circRNA	Circular RNA
CVD	Cardiovascular diseases
Cx43	Connexin-43
DE	Differential expression
I/R Injury	Ischemia/reperfusion injury
HPRT	Hypoxanthine-guanine phosphoribosyltransferase
Isl-1	Islet-1
lncRNA	Long non-coding RNA
NGS	Next-generation sequencing
pCPCspro-BNP	Porcine cardiac progenitor cellsPro-brain natriuretic peptide
qPCR	Real-time quantitative polymerase chain reaction
RNA-seq	RNA sequencing
Sca-1	Stem cells antigen-1
αSMA	Alpha smooth muscle actin
