# Supplementary material for: Novel Identified Circular Transcript of RCAN2, circ-RCAN2, Shows Deviated Expression Pattern in Pig Reperfused Infarcted Myocardium and Hypoxic Porcine Cardiac Progenitor Cells In Vitro
Source: Int J Mol Sci. 2021 Jan 30;22(3):1390. doi: 10.3390/ijms22031390 (PMC7866528; doi:10.3390/ijms22031390)
Supplement: Supplementary file 1 [file ijms-22-01390-s001.pdf]

Supplementary materials

Supplementary Table 1: Primers used for qPCR. All circRNA primers are divergent spanning the BSJ, β-Actin and HPRT primers are convergent primers.

Name	Forward	Reverse
Circ-ZNF644	CAGCCACAGAAAATGGACTTCA	TCTCGCTGCAAGAATAATCTCA
Circ-C12orf29	CTGTCTAGCCGAGCCCAAAG	GTGCAGTGCCTCTCTCAGAT
Circ-RCAN2	CTGGAACGTCACACAGTCGT	CAGTTCCTCATCTCACCCCC
Circ-SLCO5A1	TGCTAATGTTGCTGGCGATG	AAGCGCTCCCATGACATACA
β-Actin (5′-3′)	TCAACACCCCAGCCATGTAC	CTCCGGAGTCCATCACGATG
HPRT (5′-3′)	CCC AGC GTC GTG ATT AGT GA	ATC TCG AGC AAG CCG TTC AG

Supplementary Table 2: Primary and secondary antibodies used for immunofluorescence staining of pCPCs

**Primary Antibodies**				
**Type**	**Host**	**Company**	**Productnumber**	**Dilution**
Anti Sca-1	Rabbit	biorbyt	orb4622	1:200
Anti Isl-1	Rabbit	biorbyt	orb251477	1:200
Anti Cx43	Rabbit	abcam	ab11370	1:1000
Anti pro-BNP	Mouse	abcam	ab239519	1:200
Anti αSMA	Rabbit	abcam	ab5694	1:200
**Secondary Antibodies**				
**Type**	**---------**	**Company**	**Productnumber**	**Dilution**
Anti Rabbit		abcam	ab96899	1:200
Anti Mouse		biolegend	405319	1:100
